# A timely, user-friendly analysis of the mouse DNA methylome

**DOI:** 10.1016/j.xgen.2022.100153

**Published:** 2022-07-13

**Authors:** Manel Esteller

**Affiliations:** 1 Josep Carreras Leukaemia Research Institute (IJC), Badalona, Barcelona, Catalonia, Spain; 2 Centro de Investigacion Biomedica en Red Cancer (CIBERONC), 28029 Madrid, Spain; 3 Institucio Catalana de Recerca i Estudis Avançats (ICREA), Barcelona, Catalonia, Spain; 4 Physiological Sciences Department, School of Medicine and Health Sciences, University of Barcelona (UB), Barcelona, Catalonia, Spain

## Abstract

Mouse models are widely used in biomedical sciences and in epigenetic studies, yet a simple way to interrogate the mouse DNA methylation was lacking. In this issue of *Cell Genomics*, Zhou et al.^1^ describe a mouse DNA methylation microarray to simplify epigenomic analysis.

## Teaser

Mouse models are widely used in biomedical sciences and in epigenetic studies, yet a simple way to interrogate the mouse DNA methylation was lacking. In this issue of *Cell Genomics*, Zhou et al. describe a mouse DNA methylation microarray to simplify epigenomic analysis.

## Main text

DNA methylation is a chemical modification of genetic material that is involved in a myriad of cellular functions, including differentiation, aging, maintaining chromosomal structure, controlling transposable element activity, and fine-tuning gene expression in a dynamic manner. The homeostatic, but adaptive patterns of DNA methylation are altered in many human diseases, i.e., encompassing neurodegenerative disorders, metabolic diseases, and cancer. Studies of DNA methylation in cancers have shown promoter CpG island hypermethylation-associated silencing of tumor suppressor genes in the context of global genomic DNA hypomethylation. Many of the advances in this field have been possible because of reliable tools and methods allowing us to examine DNA methylation profiles in a robust and objective manner. One method is the use of DNA methylation microarrays, which are similar to widely used single nucleotide polymorphism (SNP) arrays. The gold-standard platform is the EPIC Infinium DNA methylation microarray.^2^ This tool allows for the interrogation of more than 850,000 CpG sites, a specific region of methylation, within the human genome—including coding and non-coding genes and proximal and distal regulatory regions. Subsequently, it has been utilized in thousands of studies because of its versatility and comfortability, which, respectively, enable the assessment of paraffin-embedded samples. The design of this array also permits easy sharing of raw data and database deposition. In this regard, this platform is commonly used in epigenome-wide association studies that involve large sample sets. Additionally, its use has been adopted by multi-omics initiatives such as the The Cancer Genome Atlas.

Researchers have long demanded a similar “holy grail” to characterize the mouse DNA methylome. Most mouse epigenomic studies for epigenetic markers have been carried out with whole-genome bisulphite or reduced representation bisulphite sequencing techniques. However, these approaches are expensive and time consuming and require bioinformatic analyses, limiting the availability of mouse DNA methylation data. The commercial development of the DNA methylation microarray,^1^ which has also been validated independently,^3^ provides a solution for this need within biomedical research and will boost the analyses of mouse DNA methylome studies.

**Figure 1:**
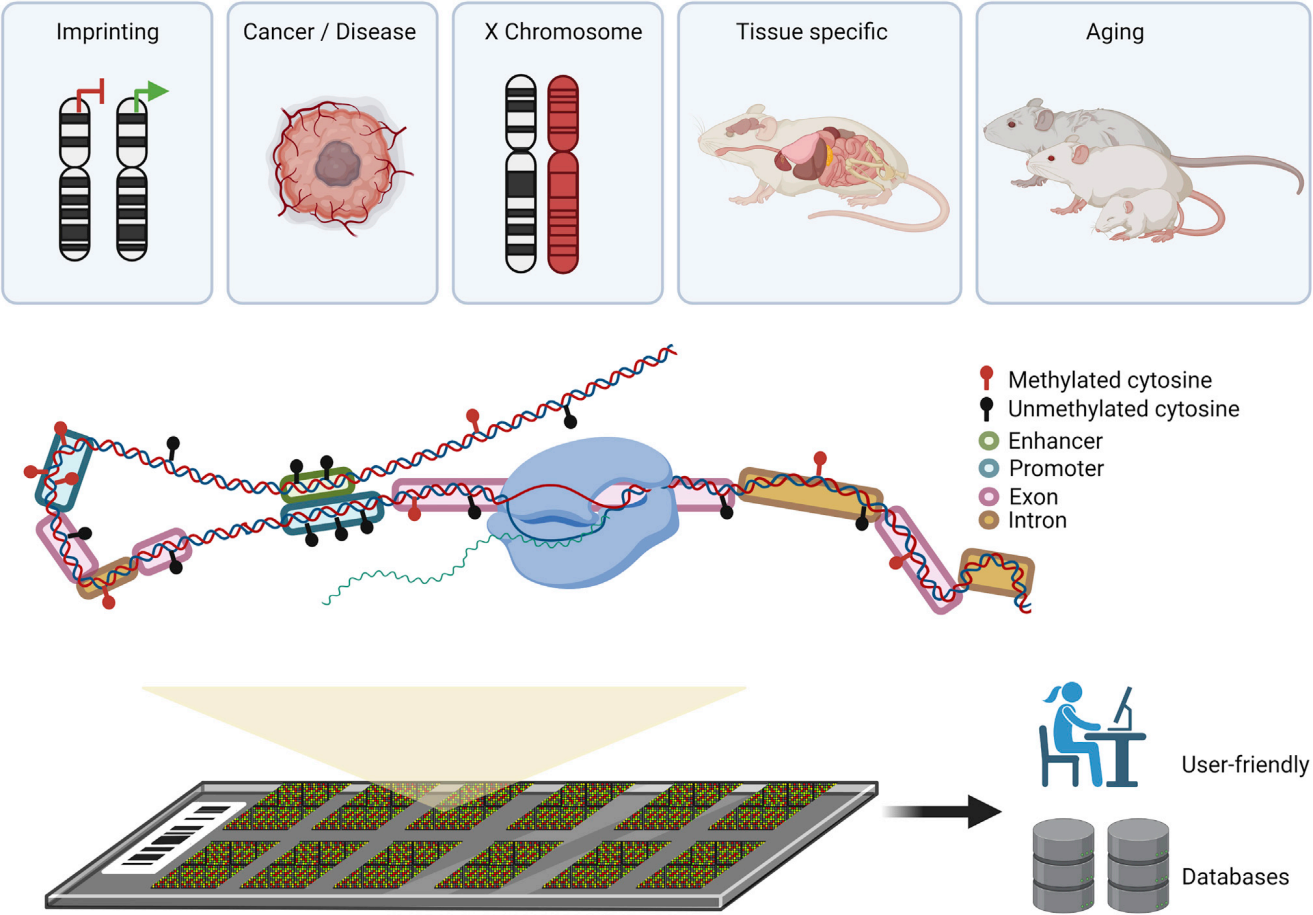
Analyzing mouse DNA methylation through a microarray Top: examples of the biological questions interrogated in Zhou et al.^1^ Bottom: an illustrative representation of CpG sites in the epigenomics platform and its user-friendly interface that allows for easy access to databases.

The mouse DNA methylation microarray introduced by Zhou et al.^1^ (Figure 1) represents an effort to capture most CpG sites and link them to functionality. Although the platform contains only 296,070 probes, less than its human counterpart, which has more than 850,000 sites, it assesses the majority of protein-coding and long non-coding RNA genes, including promoter and non-promoter CpG islands, gene body, monoallelic methylation sites, and distant regulatory regions, including super enhancers, which undergo DNA methylation shifts in cancer.^4^ Interestingly, the authors predict that beyond *Mus musculus*, this array might work for related rodent species, as rat DNA has exhibited an acceptable performance. One important aspect to be worked out is how detecting a methylated CpG site in a mouse microarray experiment could be applicable to the human DNA methylation microarray. This requires the development of bioinformatic packages to find equivalencies between the mouse and human genome and facilitate the export of data from preclinical models to patients. This is particularly important in cases where human tissue is difficult to access, such as those involving the brain,^5^^,^^6^ or in cases where studies of progressive longitudinal effects are important, such as tests of exposure to putative carcinogens.^7^

One excellent aspect of the study by Zhou et al.^1^ is its detailed explanation of the microarray composition and how it is cross-validated with other techniques to study DNA methylation (such as whole-genome bisulfate sequencing), similar to the human DNA methylation microarray.^8^ Furthermore, it indicates potential new avenues of research (Figure 1). For example, the evaluation of age-associated DNA methylation changes that show cancer-linked alterations in the *Apc*^*Min/+*^ mice enlighten the function of methylation of genomic elements, as well as shed light on epigenetic clocks,^1^ which have been proposed to be useful to predict lifespan in human studies.^9^ The capacity of this mouse methylation microarray to unveil imprinting sites, including inactivation of X-chromosome loci, tissue-type-specific DNA methylation, and backcross tracing using strain-specific SNPs,^3^ also highlights the utility of this tool.^1^

The ability to analyze a large number of mouse samples for DNA methylation profiles represents a remarkable step forward for the field of epigenetics. It means that we can now obtain a detailed readout of the components of the DNA methylation machinery and how they are affected by other biological layers, such as histone and RNA modifications or the impact of oncogenes and tumor suppressor genes in wild-type and genetically engineered mice. This tool should also be useful for analyses of screening drugs that affect the epigenome and the relevance of mouse models of human disease. For bioinformaticians, access to deposited DNA methylation data (GEO: GSE184410 and GSE196902) should generate new hypotheses and drive studies of its translation to the human epigenome. For the multi-omics “aficionado,” it is now easy to combine analyses of mouse DNA methylation data with genomics and transcriptomics results. Challenges lie ahead, such as how to apply this array to single-cell studies^10^ or studies of alternate forms of DNA methylation, such as 5′-hydroxymethylcytosine (5hmC). However, the emergence of microarray technology to elucidate mouse DNA methylomes represents a jump not only in technology but also in knowledge.
